# Microtubule-Associated Protein 1 Light Chain 3B, (LC3B) Is Necessary to Maintain Lipid-Mediated Homeostasis in the Retinal Pigment Epithelium

**DOI:** 10.3389/fncel.2018.00351

**Published:** 2018-10-08

**Authors:** Anuradha Dhingra, Brent A. Bell, Neal S. Peachey, Lauren L. Daniele, Juan Reyes-Reveles, Rachel C. Sharp, Bokkyoo Jun, Nicolas G. Bazan, Janet R. Sparrow, Hye Jin Kim, Nancy J. Philp, Kathleen Boesze-Battaglia

**Affiliations:** ^1^Department of Biochemistry, School of Dental Medicine, University of Pennsylvania, Philadelphia, PA, United States; ^2^Scheie Eye Institute, University of Pennsylvania, Philadelphia, PA, United States; ^3^Cole Eye Institute, Cleveland Clinic, Cleveland, OH, United States; ^4^Louis Stokes Cleveland VA Medical Center, Cleveland, OH, United States; ^5^Department of Ophthalmology, Cleveland Clinic Lerner College of Medicine, Case Western Reserve University, Cleveland, OH, United States; ^6^Neuroscience Center of Excellence, School of Medicine, Louisiana State University Health New Orleans, New Orleans, LA, United States; ^7^Department of Ophthalmology, Columbia University Medical Center, New York, NY, United States; ^8^Department of Pathology, Anatomy and Cell Biology, Thomas Jefferson University, Philadelphia, PA, United States

**Keywords:** LC3-associated phagocytosis (LAP), retinal pigment epithelium, lipid metabolism, oxidative stress, mouse model

## Abstract

Like other neurons, retinal cells utilize autophagic pathways to maintain cell homeostasis. The mammalian retina relies on heterophagy and selective autophagy to efficiently degrade and metabolize ingested lipids with disruption in autophagy associated degradation contributing to age related retinal disorders. The retinal pigment epithelium (RPE) supports photoreceptor cell renewal by daily phagocytosis of shed photoreceptor outer segments (OS). The daily ingestion of these lipid-rich OS imposes a constant degradative burden on these terminally differentiated cells. These cells rely on Microtubule-Associated Protein 1 Light Chain 3 (LC3) family of proteins for phagocytic clearance of the ingested OS. The LC3 family comprises of three highly homologous members, *MAP1LC3A* (LC3A), *MAP1LC3B* (LC3B), and *MAP1LC3C* (LC3C). The purpose of this study was to determine whether the LC3B isoform plays a specific role in maintaining RPE lipid homeostasis. We examined the RPE and retina of the *LC3B*^-/-^ mouse as a function of age using *in vivo* ocular imaging and electroretinography coupled with *ex vivo*, lipidomic analyses of lipid mediators, assessment of bisretinoids as well as imaging of lipid aggregates. Deletion of LC3B resulted in defects within the RPE including increased phagosome accumulation, decreased fatty acid oxidation and a subsequent increase in RPE and sub-RPE lipid deposits. Age-dependent RPE changes included elevated levels of oxidized cholesterol, deposition of 4-HNE lipid peroxidation products, bisretinoid lipofuscin accumulation, and subretinal migration of microglia, collectively likely contributing to loss of retinal function. These observations are consistent with a critical role for LC3B-dependent processes in the maintenance of normal lipid homeostasis in the aging RPE, and suggest that LC3 isoform specific disruption in autophagic processes contribute to AMD-like pathogenesis.

## Introduction

In response to stress, autophagy pathways mediate the degradation of cellular components; organelles, as well as lipids and proteins, to generate nutrients and metabolic building blocks to maintain cellular homeostasis (Dikic and Elazar, 2018; Ejlerskov et al., 2018). Autophagic processes in post-mitotic cells such as neurons, cardiac myocytes, and retinal pigment epithelium (RPE), are essential for cellular quality control, as these cells are unable to decrease their load of damaged organelles, accumulated lipids or protein aggregates through cell division (Boya et al., 2016; Liang and Sigrist, 2018). During aging, autophagy is critical to remove damaged components that would otherwise accumulate and catalyze the formation of cytotoxic molecules *in situ* under oxidative stress (Lee et al., 2012). Moreover, dysregulated autophagy contributes to accumulation of intracellular debris, inflammasome activation and cell death (Kaarniranta et al., 2013; Frost et al., 2014; Kaarniranta et al., 2017). Numerous age-related-degenerative disorders are associated with autophagic dysregulation and decreased autophagy including Parkinson’s disease and age-related macular degeneration (AMD) (Wong and Cuervo, 2010; Mitter et al., 2012; Cuervo and Wong, 2014; Ferrington et al., 2016; Golestaneh et al., 2017).

In the eye, virtually all cell types, from those comprising the cornea in the front of the eye, to the RPE providing a protective barrier for the retina at the back of the eye, rely on one or more aspects of autophagy to maintain structure and/or normal physiological function (Frost et al., 2014). Photoreceptor specific loss of a key autophagy related protein, Atg5, in the *Atg5*^Δ^*^rod^* mouse results in accumulation of photo-transduction proteins and photoreceptor degeneration (Yao et al., 2015; Zhou Z. et al., 2015). Autophagy is also a critical regulator of RPE homeostasis playing an important role in protection against oxidative stress (Ferrington et al., 2016; Kaarniranta et al., 2017; Song et al., 2017). Mutations in or loss of specific autophagy components are associated with accumulation of proteins and damaged organelles, a common characteristic of the aging RPE as well as of AMD (Mitter et al., 2012; Rodriguez-Muela et al., 2013; Boya et al., 2016; Kauppinen et al., 2016; Szatmari-Toth et al., 2016). Impaired autophagic flux is associated with disease pathology in cultured RPE from patients with AMD (Golestaneh et al., 2017). On a molecular level, oxidative stress stimulates sequestosome-1 (p62) dependent selective autophagy thereby promoting RPE cell survival (Song et al., 2017). Essential autophagy proteins, Atg7 and Atg5 (Kim et al., 2013; Zhang et al., 2017) as well as the autophagy inducer RB1CC1/FIP20 (Yao et al., 2015) are necessary to maintain visual function albeit the molecular mechanism by which this occurs varies from a role in the efficient degradation of ingested OS to the recycling of metabolic intermediates back to the photoreceptor cells. RPE specific loss of Atg5 or Atg7 predisposes RPE to retinal degeneration (Zhang et al., 2017), whereas deletion of RB1CC1/FIP20 phenocopies aspects of AMD (Yao et al., 2015).

The importance of highly regulated autophagic processes becomes clear when one considers the intimate relationship between the RPE and photoreceptor in maintaining visual function. In the central mouse retina, through a daily synchronized burst of phagocytic activity, each RPE cell can ingest the distal tips of over 200 photoreceptor cells (Volland et al., 2015). This phagocytic degradation of OS tips is a critical aspect of photoreceptor renewal and allows the post-mitotic photoreceptor cells to maintain a constant length and normal physiological function. The protein- and lipid-rich phagocytosed OS provide the RPE with a metabolic bolus of fatty acids which are oxidized providing substrate for ketogenesis (Reyes-Reveles et al., 2017). OS phagocytosis also allows for recycling of docosahexaenoic acid (DHA) (Gordon and Bazan, 1990; Rodriguez de Turco et al., 1991) and visual pigment to photoreceptors to maintain visual function (Tang et al., 2013).

The daily burst of OS phagocytosis is accompanied by an increase in LC3, levels of which fluctuate over the 12 h light/dark cycle (Yao et al., 2014; Frost et al., 2015). The RPE relies on LC3 for (1) organelle associated autophagy to maintain mitochondrial and peroxisomal health (Ferrington et al., 2016) and (2) a hybrid autophagy-phagocytosis degradative pathway termed LC3-associated phagocytosis (LAP) for degradation of OS (Kim et al., 2013; Frost et al., 2015; Martinez et al., 2015). The RPE has adapted LAP to eliminate stressors, including the daily ingestion of lipids and proteins in the form of OS tips (Kim et al., 2013; Frost et al., 2015; Reyes-Reveles et al., 2017). LAP plays a critical role in visual pigment regeneration and phagosome degradation (Kim et al., 2013; Frost et al., 2015; Muniz-Feliciano et al., 2017), with loss of LAP resulting in decreased retinal function (Kim et al., 2013) and dysregulated lipid metabolism (Reyes-Reveles et al., 2017). Recent studies in the RPE suggest that like, macrophages and dendritic cells, components of LAP specifically, RUN (RPIP8/UNC-14/NESCA) and cysteine rich domain containing beclin1 interacting protein (RUBCN), inhibit starvation induced autophagy (Martinez et al., 2015; Muniz-Feliciano et al., 2017).

Three genes encode the highly homologous, LC3 protein family members, *MAP1LC3A* (LC3A), *MAP1LC3B* (LC3B) and *MAP1LC3C* (LC3C) (Klionsky et al., 2016). Although mammalian LC3 isoforms were predicted to be functionally redundant, some studies suggest isoform specificity in selective autophagy (Shpilka et al., 2011; Maruyama et al., 2014; Koukourakis et al., 2015). Whether the LC3 isoforms are functionally redundant or play a specialized role in maintaining RPE homeostasis is completely unknown and is the focus of our studies. We show that loss of LC3B results in decreased RPE-OS phagosome clearance and a concomitant decrease in fatty acid oxidation and ketogenesis. In these studies, we examine the long-term consequences of LC3B ablation on RPE function. We measure the development of an array of phenotypes found in age-related retinal disease, including, accumulation of RPE and sub-RPE lipid deposits likely predisposing the RPE to an enhanced pro-inflammatory micro-environment, as well as decline in RPE and retinal cell function.

## Materials and Methods

### Animals

C57BL6/J (WT) mice and the *LC3B*^-/-^ mouse line (strain name: B6;129P2-Map1Lc3b^tm1Mrab^/J; stock # 009336, (Cann et al., 2008) were purchased from Jackson Laboratory (Bar Harbor, ME). The *LC3B*^-/-^ mice were backcrossed for at least five generations onto a C57BL6/J background. The *LC3B*^-/-^ and the WT mice were confirmed to be free of the rd8 mutation by genomic DNA PCR using primers as described in Chang et al. (2013). Maintenance of mouse colonies and all experiments involving animals were as described previously (Frost et al., 2015, 2017). Mice were housed under standard cyclic light conditions: 12-h light/12-h dark and fed *ad libitum*, with both female and male mice used in these studies. All procedures involving animals were approved by the Institutional Animal Care and Use Committees of the University of Pennsylvania and of Cleveland Clinic, and were in accordance with the Association for Research in Vision and Ophthalmology guidelines for use of animals in research.

### Antibodies

Commercially available primary antibodies used were: mouse anti-β-Actin (A2228; Sigma-Aldrich, St. Louis, MO, United States), rabbit anti-LC3A (ab62720), rabbit anti-HMGCS2 (ab137043, EPR8642) rabbit anti-4 Hydroxynonenal (4-HNE) antibody (ab46545) from Abcam (Cambridge, MA, United States), rabbit anti-LC3B (ab48394) from Abcam, rabbit anti-LC3B (D11) from Cell Signaling Technology (Danvers, MA, United States), rabbit anti-Iba1 (019-19741) from Wako Chemicals, Japan. Secondary antibodies used were: goat anti-mouse and goat anti-rabbit horseradish peroxidase (HRP)-conjugated antibodies (Thermo Fisher Scientific), goat anti-mouse and anti-rabbit IgG Alexa Fluor 488/594/647 conjugates (Invitrogen).

### RT PCR

Total RNA from mouse retina or RPE was isolated with a Nucleospin RNA II kit (Macherey-Nagel, Bethlehem, PA, United States) and cDNA was synthesized with a high-capacity cDNA reverse transcription kit (Applied Biosystems, Foster City, CA, United States). Quantitative real-time PCR was performed with a Power SYBR Green kit (Applied Biosystems) as described (Nikonov et al., 2013). *Gapdh* was used as a reference gene for normalization. There is no gene corresponding to human *MAP1LC3C* in mouse, hence mouse *Map1lc3a* and *Map1lc3b* specific PCR primers were used:

Map1lc3a (NM_025735.3)
Forward: 5′-GACCGCTGTAAGGAGGTGC-3′
Reverse: 5′-CTTGACCAACTCGCTCATGTTA-3′
Map1lc3b (NM_026160.4)
Forward: 5′-TTATAGAGCGATACAAGGGGGAG-3′
Reverse: 5′-CGCCGTCTGATTATCTTGATGAG-3′
Gapdh
Forward: 5′-CCCACTAACATCAAATGGGG-3′
Reverse: 5′-CCTTCCACAATGCCAAAGTT-3′

### Immunoblotting

Cleared RPE and retinal lysates were prepared in RIPA buffer with 1% protease inhibitor mixture (Sigma; P8340) and 2% phosphatase inhibitor mixture 2 (Sigma; P5726). 10–15 μg of protein was separated on 12% Bis-Tris-PAGE (Invitrogen) under reducing conditions and transferred to PVDF membranes (Millipore, Billerica, MA, United States). Membranes were blocked with 5% milk in PBS, 0.1% Tween-20 for 1 h at room temperature and incubated with primary antibodies for anti-HMGCS2 (1:1,000), anti-β-actin (1:5,000), anti-LC3A (1:1,000), or anti-LC3B (1:1,500) overnight at 4°C. Membranes were washed and incubated with goat anti-rabbit (1:3,000) or goat anti-mouse (1:3,000) HRP-conjugated secondary antibodies for 1 h at room temperature. The blots were developed using ECL SuperSignal^®^ West Dura extended duration substrate (Thermo Scientific) and captured on Odyssey Fc (Licor) and quantified as described (Reyes-Reveles et al., 2017).

### Immunostaining

Immunostaining was performed on mouse retinal cryosections or RPE/choroid flat-mounts. Mice were anesthetized, and the eyes were enucleated, incised just below the ora serrata and fixed in 4% paraformaldehyde in PBS (pH 7.4) for 18h at 4°C followed by PBS wash to remove fixative. The eyes were then cryoprotected in 30% sucrose, embedded in OCT and stored at -80°C. Cryosections were prepared by radially sectioning the blocks at 10 μm thickness. The RPE/choroid flat-mounts were prepared by separating the retina from the RPE followed by fixation in 4% PFA for 30 min at room temp. The sections or flat-mounts were permeabilized and blocked in blocking solution containing 5% BSA and 0.2% Triton X-100 in PBS (PBST) at 37°C for 1 h, incubated with the primary antibody diluted in blocking solution (1:500 for anti Iba1 or 1:50 for rabbit 4-HNE) at 4°C overnight, washed three times with PBST, incubated in appropriate secondary antibodies conjugated to Alexa Fluor dyes (Invitrogen, 1:1,000) and Hoechst 33258 (1:10,000) at 37°C for 1 h and washed three times. In some of the flat-mount staining experiments, Alexa Fluor 647 Phalloidin (1 Unit/200 μl) (Invitrogen) was included in the secondary antibody step. For no antibody control, the primary antibody step was eliminated. Sections were mounted in Cytoseal mounting medium (Electron Microscopy Sciences, Hatfield, PA). Images were captured on a Nikon A1R laser scanning confocal microscope with a 10 × (NA 0.45) or 20 × (NA 0.75) dry objectives or a PLAN APO VC 60× water (NA 1.2) objective at 18°C, and the data were analyzed using Nikon Elements AR 4.30.01 software.

### Quantification of Phagosomes *in vivo*

Phagosome were counted in mouse retinal ultrathin sections as described (Damek-Poprawa et al., 2009). Briefly, WT and *LC3B*^-/-^ mice (∼4 months old) kept under same light cycle and were euthanized at -60 min, 30 min, 1 h, 7 h, 13 h after light onset. Fixed eyecups were embedded in Epon and ultrathin sections were taken from blocks containing well aligned photoreceptor cells. For each eye, 15 areas of 120 μm^2^ were scanned at 5000× magnification, and phagosomes were counted directly on a Jeol-1010 transmission electron microscope screen. The count was done under masked conditions so that the genotype of the specimen was unknown to the person performing the analysis. Counts were performed by two different individuals, each counting 15 different areas per mouse. Structures with diameters of at least 75% of the mean diameter of ROS were counted as phagosomes only if they were present in the RPE cytoplasm and contained lamellar membranes similar to intact photoreceptor outer segment disks (Nandrot et al., 2004).

### Staining for Cholesterol and Neutral Lipids

BODIPY^TM493/503^(4,4-Difluoro-1,3,5,7,8-Pentamethyl-4-Bora- 3a,4a-Diaza-s-Indacene, Molecular Probes, Eugene, OR, United States; D3922) was used to visualize neutral lipid-rich deposits. BODIPY^TM493/503^ stock solution (0.5 mg/ml) was prepared in 100% ethanol and diluted in 1×PBS to 10 μg/ml before the experiment. Retinal cryosections were air dried, rehydrated in 1× PBS and incubated in BODIPY^TM493/503^ for 1 h at RT in the dark, followed by nuclear staining (Hoechst 33298) and three, 5-min PBS washes. Images analysis was performed by NIS Elements AR 4.30.01 software. Briefly, after background subtraction, a region of interest (ROI) was drawn across the RPE, the mean ROI intensity was obtained for each image and averaged for different images for each experiment. The intensity measure for the *LC3B*^-/-^ was normalized relative to WT measurement for each experiment and averaged (*N* = 3 sets). For puncta count, an intensity threshold was applied for the ROI and binary image was then analyzed for the number of puncta. For filipin staining, retinal cryosections were incubated in 100 μg/ml Filipin (from 1 mg/ml stock in DMSO, Sigma F9765) for 1 h at RT in dark, followed by three, 5-min PBS washes. Slides were mounted in cytoseal and imaged as described above.

### β-Hydroxybutyrate and Citrate Synthase Assays

WT and *LC3B*^-/-^ (12–15 months) mice were sacrificed at 7 a.m (light onset) with at least three mice of each genotype analyzed. RPE explants were isolated, placed in a 96-well plate with 170 μl of Ringer’s solution and incubated at 37°C, 5% CO_2_. 2 h after harvest, 100 and 50 μl of the Ringer’s solution was collected for β-hydroxybutyrate (β-HB) determination. β-HB release was measured from the explants using the β-hydroxybutyrate LiquiColor kit (Stanbio, Boerne, TX; catalog no. 2440-058) (Reyes-Reveles et al., 2017). RPE cell lysates were then prepared from the explants for immunoblotting. Mitochondrial health was measured as citrate synthase activity using Mitocheck citrate synthase activity assay (Cat. # 701040, Cayman Chemical, Ann Arbor, MI, United States) as described (Iacovelli et al., 2016). This assay is based on an absorbance readout of the SH-CoA released when citrate synthase catalyzes condensation of oxaloacetate and acetyl CoA to form citrate. The reaction was initiated with the addition of oxaloacetate to reaction wells containing RPE lysates typically diluted 50×. Absorbance at 412 nm as a function of time was measured for lysate samples, positive controls (using a known amount of enzyme), as well as negative controls (with either no enzyme or no enzyme and no oxaloacetate). Specific activity was determined by dividing the reaction rate by lysate protein concentration.

### Lipid Extraction and Mass Spectrophotometry

Lipids were extracted from samples of RPE explants from 18 to 20 months old mice sacrificed at 10 am, using a modified Folch method (Folch et al., 1957). Briefly, a glass homogenizer was used to homogenize the tissues in 3 ml of cold methanol to which an internal standard mixture (5 ngs of LTB4-d4, PGD2-d4, 15-HETE-d8, EPA-d5, and 25 ng of AA-d8) was added. After adding 6 ml of CHCl_3_, the sample was vortexed and sonicated on ice for 30 min. Upper phase was removed after the addition of 2 ml of pH 3.5 0.1NHCl which caused the phase separation. Sample was hydrolyzed to measure total fatty acid (DHA, AA, and EPA) composition as follows: sample in 100 μl MeOH was added to 48 μl 1N NaOH and 52 μl H_2_O, and incubated in a water bath at 42°C for 3 h. After addition of 400 μl H_2_O the pH of the sample was adjusted to 3.5 ∼ 4.0 with 0.1N HCl. Ethyl acetate (2 ml) was added to facilitate a phase separation. After centrifugation, the upper phase was transferred to another container and Ethyl acetate extraction repeated and upper phases combined. The upper phase was dried under N_2_ and the sample was resuspended in 30 μl of MeO:H_2_O = 1:1 for mass spec. Xevo TQ-S equipped with Acquity I Class UPLC (Waters Corporation, Milford, MA, United States) was used for lipidomics. Acquity UPLC HSS T3 1.8 μm 2.1 mm × 50 mm Column was used for fatty acids and their derivatives. 45% of solvent A (H_2_O + 0.01% acetic acid) and 55% of solvent B (MeOH + 0.01% acetic acid) with 0.4 ml/min flow was used initially and gradient to 15% of A for the first 10 min, then gradient to 2% of solvent A at 18 min. 2% of solvent A ran for 25 min, and gradient back to 45% of A for a 30 min re-equilibration. The capillary voltage was -2.5 kV, desolvation temperature at 60°C, desolvation gas flow at 1100 L/Hr, cone gas at 150 L/Hr, and nebulizer pressure at 7.0 Bar with the source temperature at 150°C. MassLynx 4.1 software was used for operation and data recording.

### Ocular Imaging

Confocal scanning laser ophthalmoscopy (SLO, Model HRA2, Heidelberg Engineering) and Spectral-domain optical coherence tomography (SDOCT, Model SDOIS, Leica Bioptigen) were performed as previously described (Bonilha et al., 2015). Briefly, mice (3.5, 12, or 21 months old) were anesthetized with an IP injection 68–69 mg/kg of sodium pentobarbital. Pupils were dilated with 1 μl of 0.5% tropicamide/phenylephrine combination drops. Topical anesthesia was achieved using 0.5% Proparacaine. Eyes were kept hydrated using Systane Ultra Hydrating tears and ocular eye shields (Bell et al., 2014).

The SLO images were collected with the camera focus trained on the RPE/choroid interface. All five standard SLO modes were collected including IR reflectance (IR-SLO), IR and Red Free Dark field (IR- and RFDF), and Blue and IR autofluorescence (AF- and IR-SLO). Images were obtained with the optic disk centrally located within the field of view (FOV) using a 55° wide-field lens. Images were captured with active normalization enabled and averaged 25 times using the Automatic real-time averaging (ART) feature. IRDF images were selected from the pool of imaging data obtained for SLO image analysis. Images were exported as TIFF files with LZW compression and imported into ImageJ v.1.48V (Schneider et al., 2012) as RGB and converted to 8-bit grayscale. The scale bar in the lower left corner of the image panel was removed, then an automated Renyi Entropy threshold, using the color “red” and “Dark background” enabled, was applied to the image, and an area fraction measurement was obtained (Analyze > Measure) (Kapur et al., 1985). All mice were analyzed in this same manner.

Spectral-domain optical coherence tomography images were collected using a radial volume scan set to 0 and 90°. With the optic disk centrally located with the FOV, this orthogonal scan configuration permitted collection of two B-scans sets from the horizontal and vertical meridians. Each B-scans set (10–15 frames @ 0° and 90°) was collected using1000 A-scans/B-scan. Radial scans were converted to AVI files and exported to ImageJ with an axial scale of 1 μm/pixel. In ImageJ, each set of B-scans was co-registered and averaged using StackReg/TurboReg plug-ins (Thevenaz et al., 1998). Mean ONL thickness was measured from each regional quadrant (superior, inferior, temporal, and nasal) halfway from the optic disk to the edge of the image using the Straight Line tool. ONL thickness from the superior, inferior, temporal and nasal regions was averaged to obtain a mean for each eye. To capture the presence and/or absence of outer retinal pathology, the Freehand Line tool was employed to trace of the hyper-reflective outer retina lamina band that includes interface between photoreceptor layer outer segments and RPE.

All data were analyzed and graphed (Mean ± SEM) in GraphPad Prism 6. Statistical significance was determined using an Ordinary One-way ANOVA and uncorrected Fisher Least Significant Difference (LSD) test. *P* ≤ 0.05 were considered significant.

### Electroretinograms

WT and *LC3B*^-/-^ mice between the ages of 14 and 20 months were analyzed. After overnight dark adaptation, mice were anesthetized with ketamine (80 mg/kg) and xylazine (16 mg/kg) diluted in 0.9% saline. The pupils were dilated with 1% tropicamide, 2.5% phenylephrine HCl, and 1% cyclopentolate eye drops. The corneal surface was anesthetized with 1% proparacaine HCl eye drops. ERGs were recorded using three electrodes. The active lead was a thin stainless-steel wire, coiled at the end into a loop and wetted with 1% carboxymethylcellulose. The reference and ground leads were needle electrodes inserted in the cheek or tail, respectively. ERGs were recorded to strobe flash stimuli presented in an LKC (Gaithersburg, MD, United States) ganzfeld. Stimuli were first presented to the dark-adapted eye (-3.6 to 2.1 log cd s/m^2^). After the dark-adapted series was completed, a steady 20 cd/m^2^ background field was presented in the ganzfeld. After a 5-min light adaptation period, a light-adapted series of responses were recorded stimuli (-0.8 to 2.1 log cd s/m^2^). Responses were band-pass amplified (0.03–1000 Hz), averaged, and stored using an LKC UTAS E-3000 signal averaging system. In most cases, we used a 500 ms recording epoch. To examine the ERG c-wave, we recorded responses for 4 s duration (Kinoshita and Peachey, 2018).

The amplitude of the a-wave was measured at 8 ms after flash onset from the pre-stimulus baseline. The leading edge of the a-wave to a 1.4 log cd s/m^2^ flash was measured using the equation (1):

$$\begin{array}{c} P3(i,t)\;=\;(1-exp(-iA{\left(t-td\right)}^{2}))RmP3 \end{array}$$

Where RmP3 is the maximum response amplitude, *A* is a measure of sensitivity, and td is the delay in phototransduction (Breton et al., 1994).

The amplitude of the b-wave was measured from the a-wave trough to the b-wave peak. The response function relating b-wave amplitude was fitted using the Michaelis–Menten equation (2) (Fulton and Rushton, 1978):

$$\begin{array}{c} {R/R}_{\max}= L/(L+K) (Fulton and Rushton, 1978) \end{array}$$

where *R* is the amplitude of the a- or b-wave; *R*_max_ is the maximum amplitude of the a- or b-wave; *L* is the flash energy (log cd s/m^2^); and *K* is the flash energy that elicits an amplitude of half *R*_max_ (half-saturation coefficient). Luminance-response functions for the major ERG components were compared between WT and KO mice using two-way repeated measures analysis of variance (ANOVA). Curve fit parameters were analyzed using the Students’ *t*-test.

### Tissue Extraction and Quantitative UPLC Analysis of Mouse Eyecups

Mouse eyecups (2–3 eyes/sample as indicated) were homogenized in DPBS using a tissue grinder in the presence of chloroform/methanol (1:1). Subsequently, the sample was extracted three times with addition of chloroform and centrifuged at 3220g for 10 min. After passing through a cotton filter, the extract was concentrated by evaporation of solvent under argon gas and was dissolved in ethanol. The samples were examined by UPLC (Waters Acquity UPLC system; Waters, Milford, MA, United States) using a phenyl column (Waters ACQUITY UPLC^®^ BEH Phenyl; 1.7 μm, 2.1 mm × 100 mm) with a mobile phase of acetonitrile/water (1:1) and isopropanol/acetonitrile (9:1) both with 0.1% formic acid (0–50 min, 100–55% acetonitrile/ methanol in isopropanol/acetonitrile; 50–110 min, 55–35% acetonitrile/methanol in isopropanol/acetonitrile; flow rate of 0.2 mL/minute); injection volume, 10 μL. UV absorbance peaks were identified by comparison with synthetic standards (bisretinoids; A2GPE and atRALdiPE). The quantity of bisretinoids in each sample calculated from peak areas using Empower software (Waters, Milford, MA, United States).

### Lipid Peroxidation

WT or *LC3B*^-/-^ mice were sacrificed at 10 am (3 h after light onset), with at least three mice of each genotype analyzed. RPE explants were isolated as described (Reyes-Reveles et al., 2017) and immediately prepared for 4-HNE analysis. Cleared RPE cell homogenates obtained by centrifugation at 2000 × *g* for 3 min were used in twofold series dilutions. Samples were analyzed using 4-HNE- ELISA kit from Cell Biolabs, San Diego, CA, United States according to the manufacturer’s directions using a Multiskan MCC plate reader (Thermo Fisher Scientific, Waltham, MA, United States). Protein was quantified using Bradford reagent (Thermo Fisher). Data represents mean ± SEM, *N* = 6, 2 eyes each from 3 individual mice.

### Measurement of Sterols

Cholesterol and 7-ketocholesterol was analyzed as described (Rodriguez, 2004). Briefly, flash frozen RPE explants isolated from *LC3B*^-/-^ or WT mice were lyophilized. Rather than saponifying the tissue, which tends to variably degrade 7KCh, the RPE was directly extracted with organic solvents with 1β-sitosterol (5 nmol) serving as an internal standard and dried under argon as previously described (Bligh and Dyer, 1959). Extracted lipids were re-dissolved in methanol and analyzed directly by high pressure liquid chromatography and mass spectroscopy (LCMS) (Moreira et al., 2009).

## Results

### Absence of LC3B Leads to Retinal and RPE Abnormalities

**FIGURE 1:**
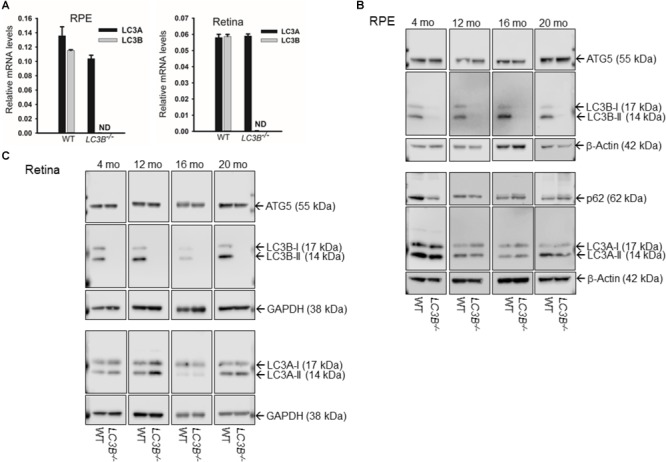
**(A)** LC3A and **(B)** transcript levels by qRT-PCR in RPE (left) and retina (right) for WT and *LC3B^-/-^* mice as indicated. Results are mean (±SD) of three individual experiments (4 months). ND, not-detectable. **(B)** Representative immunoblots showing expression of ATG5, LC3B, P62/SQSTM1, and LC3A proteins in RPE from 4, 12, 16, and 20 months old mice. **(C)** Representative immunoblots showing expression of ATG5, LC3B, and LC3A proteins in retina from WT and *LC3B^-/-^* mice of different ages as indicated. The mice were sacrificed between 3 and 4 h after light onset.

We examined the role of LC3B in retinal homeostasis using an *LC3B*^-/-^ mouse, in which deletion of exons III and IV of *Map1lc3b* results in a null allele (Cann et al., 2008). As expected, LC3B transcript (**Figure 1A**) and LC3B protein were absent in the *LC3B*^-/-^ mouse RPE (**Figure 1B** and **Supplementary Figure S1A**) and retina (**Figure 1C**); moreover, there was no significant up-regulation of LC3A mRNA or protein in the RPE or retina (**Figures 1A–C**), consistent with previous studies using mouse embryonic fibroblasts (Cann et al., 2008). No significant change in the levels of ATG5, an essential autophagy related protein and p62, an LC3 adaptor protein involved in selective autophagy (**Figure 1C**) was detected in *L3CB*^-/-^ RPE relative to the WT for different age groups analyzed (4, 12, 16, and 20 months).

**FIGURE 2:**
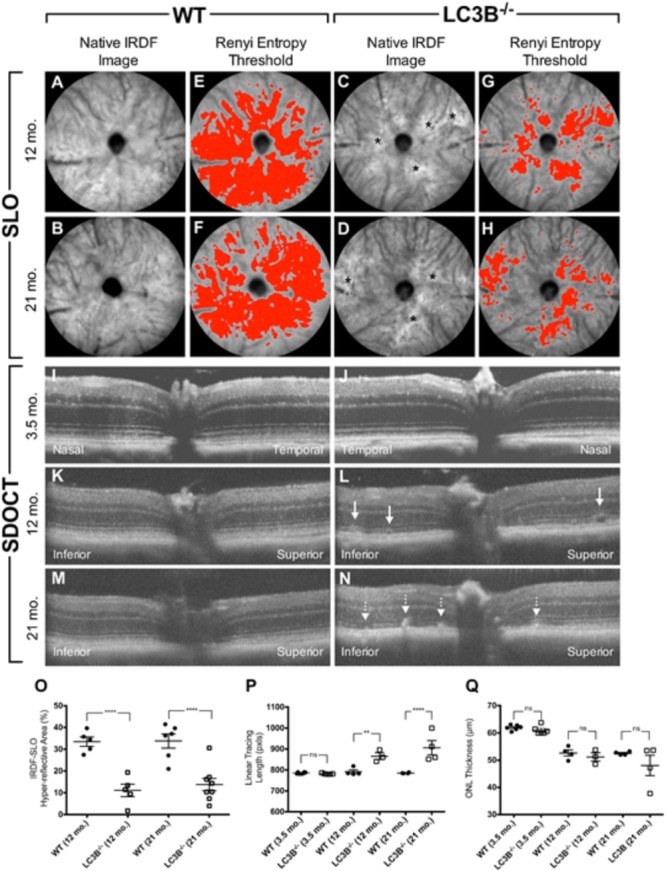
Representative SLO **(A–H)** and SDOCT **(I–N)** images from young (3.5 months old) or aged (12 and 21 months old) *L3CB^-/-^* mice compared to age-matched WT controls. Quantified imaging results for hyper-reflective features observed by IRDF-SLO **(O)** and SDOCT **(P,Q)**. Native IRDF-SLO images in fundus architecture in WT **(A,B)** and *L3CB^-/-^* **(C,D)**, note irregular white patches, black asterisks in the *LC3B^-/-^*; A Renyi threshold applied to the SLO images captures the level of homogeneity, or inhomogeneity, in the hyper-reflective area for WT **(E,F)** and *L3CB^-/-^* **(G,H)** mice, respectively. SD-OCT: WT **(I,K,M)** and 3.5 months old *LC3B^-/-^* **(J)** have normal architecture whereas the older *L3CB^-/-^* **(L–N)** examples have localized disruptions within the photoreceptor-attributable layer, immediately adjacent to the RPE. Sub-retinal vacuoles (**L**; solid line arrows) and hyper-reflective pathology (**N**; dotted line arrows) observed in the photoreceptor layer of 12 and 21 months old *L3CB^-/-^* examples relative to WT controls **(K,M)**. The linear tracing length analysis **(P)** captures the presence or absence of suspected abnormal pathology in the photoreceptor/RPE region between *L3CB^-/-^* and WT. SDOCT image depth is 0.42 mm **(I–N)**. Graphical data **(O–Q)** shown as mean ± SEM: ns = *p* > 0.05, ^∗^*p* < 0.05, ^∗∗^*p* < 0.01, ^∗∗∗^*p* < 0.001, and ^∗∗∗∗^*p* < 0.0001. *N* = 6 (3.5 months WT and *LC3B^-/-^*), 4 (12 months WT), 3 (12 months *LC3B^-/-^*), and 4 (21 months WT and *LC3B^-/-^*).

We examined the retina and RPE of *LC3B*^-/-^ mice using, non-invasive imaging modalities, IRDF-SLO and SDOCT. Atypical retinal features were distinguishable in older *L3CB*^-/-^ mice vs. age-matched WT controls using both imaging modalities (**Figure 2**). These observations were not found in young mice (∼3.5 months) using either SLO (images not shown) or SDOCT (**Figures 2I,J**). Representative IRDF-SLO images collected from the RPE-choroid complex of *L3CB*^-/-^ mice show hyper-reflective regions (**Figures 2C,D**) that were on average, brighter than the typical, homogenous background observed in WT mice (**Figures 2A,B**). The dark circles and numerous dark streaks throughout the images are the optic disk and the arteries and vasculature within the choroidal plexus, respectively. WT mice in general have a uniform spatial intensity distribution across the observable FOV (**Figures 2A,B**). The major limitation for obtaining “complete” uniformity can be attributed to the fact that the retina is a curved entity and obtaining full-field, aberration-free images with uniform intensity is difficult, if not practically impossible as the reflective signal strength falls off radially from the center of the image. In contrast to WT controls, *L3CB*^-/-^ mice exhibited an atypical spatial intensity distribution, as swathes of retina appear brighter than immediately adjacent, normal-appearing regions (**Figures 2C,D**). This atypical distribution is discernable by eye and is suggestive of retinal pathology in the *L3CB*^-/-^ mice. Although sufficient contrast is available for manual quantification, to better accentuate the differences between KO and WT we include a more objective analysis by employing Renyi entropy threshold, an automated threshold tool in ImageJ (Kapur et al., 1985). The spatial intensity distribution is quite uniform for WT and encompasses nearly 30–40% of the entire image (**Figures 2E,F**); whereas in *L3CB*^-/-^ mice the uniformity is substantially reduced, corresponding to a much smaller fraction (∼10%) of the image (**Figures 2G,H**). At both 12 and 21 months, *LC3B*^-/-^ mice had hyper-reflective changes that resulted in a significant overall reduction in threshold area relative to the controls (**Figure 2O**), suggesting abnormal retinal pathology near the RPE in the *LC3B*^-/-^.mice. Moreover, it suggests that these changes are more punctate and above the typical background variations observed in WT mice. No significant differences in IRDF hyper-reflective area were observed between 12 and 21 months in either *L3CB*^-/-^ or WT controls. In addition, such changes were not apparent in a comparison of 3.5 months old WT and *L3CB*^-/-^ mice (data not shown).

Representative OCT B-scans from the vertical meridian of WT and KO mice are shown in **Figures 2I–N**. Qualitatively, young mice comparisons have no discernable difference in retinal lamina architecture (**Figures 2I** vs. 2J). The qualitative comparison shows hyper-reflective changes in the outer retina in *L3CB*^-/-^ mice compared to age-matched WT controls (**Figures 2L,N** vs. 2K,M). Notably, the outer retina banding between outer nuclear layer (ONL), inner segment (IS), outer segment (OS), RPE and choroid (Ch) have all undergone localized disruption relative to controls. These observations were quantitatively compared using a linear tracing of the dark to bright banding adjacent to the outer segments/RPE. This approach allows us to follow the contour of abnormal SDOCT pathology along the length of viewable retina. The hyper-reflective pathology observed in the outer retina of *L3CB*^-/-^ mice was determined as significant relative to WT at both 12 and 21 months (**Figure 2P**).

**FIGURE 3:**
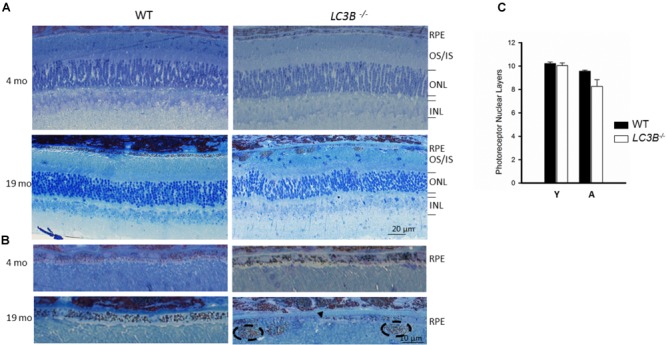
Representative images for semithin sections from 4 and 19 months old WT and *L3CB^-/-^* stained with 1% Toluidine blue showing retinal layers **(A)** and RPE **(B)**. Older *L3CB^-/-^* shows abnormal features in the RPE/sub-RPE space including hypopigmentation (arrow-head) and hyperpigmentation (dotted oval). **(C)** Bar graph showing average number of nuclear layers in the ONL (outer nuclear layer) for young (Y, 3–4 months) and aged (A, 16–19 months) mice. OS/IS, outer segment/inner segment; INL, inner nuclear layer.

To determine if outer retinal abnormalities correlated with photoreceptor degeneration, ONL thickness was measured from the same SDOCT B-scans. Although the analysis demonstrated that *L3CB*^-/-^ mice tended to have slightly thinner ONL, with one 21 months old mouse having 30% thinning, the mean differences were found to be insignificant relative to controls (**Figure 2Q**) at each age group. We further analyzed the retinal structure by counting the number of nuclei in the ONL (outer nuclear layers) for young and aged WT and *L3CB*^-/-^ retinal images (**Figures 3A,B**). While, there was no difference in the number of nuclear layers between young WT and *L3CB*^-/-^ mice, there was a slight decrease (albeit not significant) in the number of nuclear layers for the aged (16–19 months) *L3CB*^-/-^ mice relative to control (**Figure 3C**). Besides this, there appear to be several hyper and hypo-pigmented areas in the RPE from older *LC3B*^-/-^ and migration of cells (possibly microglia) in the subretinal space (**Figures 3A,B**). Collectively, these studies suggest age-dependent changes in the *L3CB*^-/-^ RPE structure and sub-retinal space with relatively intact retina.

### Slowed Phagosome Degradation, Dysregulation of Lipid Metabolism and Lipid Accumulation

**FIGURE 4:**
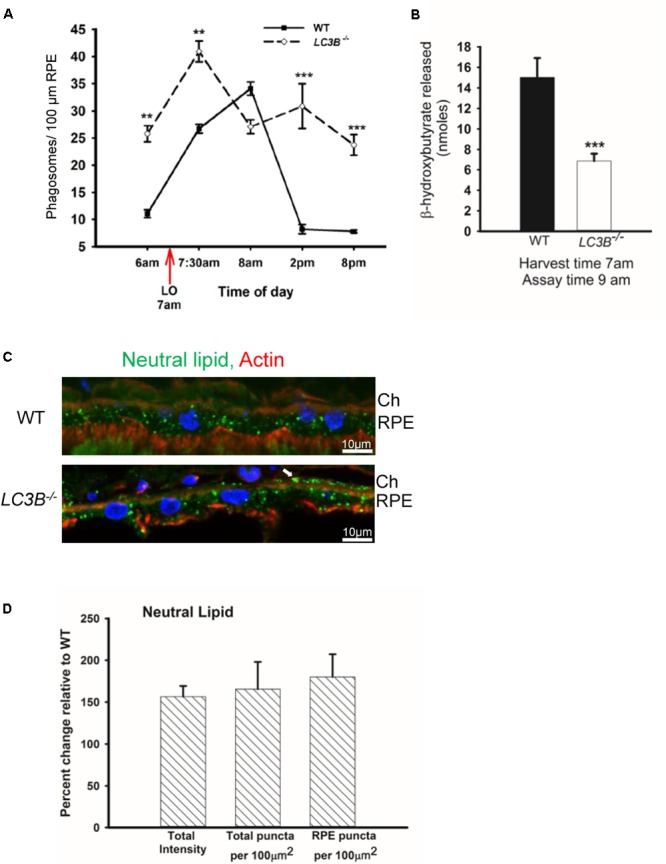
Loss of LC3B results in phagosome and neutral lipid accumulation. **(A)** Phagosomes per 100 μm of RPE length in WT and *LC3B^-/-^* mice (∼4 months old) at different times of day relative to light onset (LO is at 7 am); lights were turned off at 7 pm. Phagosomes were counted under masked conditions, in duplicate on 15 different areas per mouse. Values are mean ± SEM of two eyes, 15 regions per eye. ^∗∗^*p* < 0.005, and ^∗∗∗^*p* < 0.002. **(B)** β-HB released from RPE explant harvested at light onset (7 am) and assayed at 9 am in WT and *LC3B^-/-^* mice (12–15 months old). Values are mean ± SEM from 4 individual mice. ^∗∗∗^*p* < 0.002. **(C)** Representative confocal image of neutral lipid deposits detected by staining with BODIPY^493/503^ in WT and *LC3B^-/-^* RPE (18 months) nuclei (blue) stained with HOECHST, Ch – choroid. **(D)** Intensity of BODIPY^493/503^ staining was quantified and puncta localized as indicated. Values are mean ± SEM from 3 individual mice (∼18 months), ^∗^*p* < 0.050.

The SLO and SDOCT imaging studies suggested alterations in RPE, therefore we asked if a primary RPE function – the daily ingestion and degradation of shed photoreceptor outer segment tips is altered. To determine if LC3B is necessary in synchronized phagocytosis of shed OSs we counted the number of phagosomes in ultrathin sections of RPE from *LC3B*^-/-^ and WT mice fixed at different times of day. In WT RPE, phagosome numbers reached a sharp peak within an hour of lights on (**Figure 4A**) (LaVail, 1980; Gibbs et al., 2003; Nandrot et al., 2004). In the *LC3B*^-/-^ RPE, phagosome numbers were already elevated before light onset, and then rose only moderately after light onset. Elevation in phagosome numbers in *LC3B*^-/-^ RPE was evident for up to 14 h and never returned to the WT baseline values prior to the next burst of OS uptake (**Figure 4A**). Representative electron micrographs at 7:30 am and 2 pm are shown in **Supplementary Figure S2** for WT and *LC3B*^-/-^ mice.

In RPE, the lipid-rich OS phagosomes provide substrate for fatty acid oxidation (FAO) and ketogenesis (Reyes-Reveles et al., 2017), processes that supply metabolic intermediates for the outer retina (Adijanto et al., 2014). β-hydroxybutyrate (β-HB) is preferentially transported across the apical membrane of the RPE and is utilized by photoreceptor cells (Adijanto et al., 2014). In WT mice, 15.0 ± 1.89 nmoles of β-HB was released by 9 am; in contrast, the *LC3B*^-/-^ RPE released only 6.87 ± 0.71 nmoles of β-HB (**Figure 4B**). This decrease is not due to altered levels of mitochondrial HMGCS2, the rate limiting enzyme in ketogenesis, since both *LC3B*^-/-^ and WT RPE express equivalent levels of HMGCS2 (**Supplementary Figures S1B,C**). Moreover, mitochondrial function based on citrate synthase activity was unaltered in the absence of LC3B (**Supplementary Figure S1D**). Thus, we hypothesize that the 50% decrease in β-HB is likely due to the reduced availability of free fatty acids from ingested OS phospholipids (Reyes-Reveles et al., 2017).

Slowed phagosome degradation coupled with the decrease in fatty acid oxidation (as reflected in β-HB release) could result in an accumulation of lipid in the RPE. We investigated the levels of intracellular RPE lipids in older *LC3B*^-/-^ mice (∼18 months old). *LC3B*^-/-^ RPE exhibited an abundance of neutral lipid droplets, detected as BODIPY^TM^ positive puncta, compared to WT; in the *LC3B*^-/-^ neutral lipid containing structures were found not only in RPE but also at RPE-choroid interface (**Figure 4C**, white arrow). There was a 1.6-fold increase in the intensity of BODIPY^TM493/503^ in *LC3B*^-/-^ mouse RPE compared to WT (**Figure 4D**), with numbers of lipid puncta ∼185% higher in *LC3B*^-/-^ RPE compared to WT (**Figure 4D**). Lipid debris increased as the mice aged, with virtually no BODIPY^TM493/503^ positive puncta in RPE of young animals (2 months old) and an accumulation of RPE and sub-RPE lipid deposits by 18 months (**Supplementary Figure S3A**). Cholesterol is a major component of neutral lipid droplets. We observed an increase in vesicular binding of filipin, consistent with elevated intracellular cholesterol in *LC3B*^-/-^ RPE. Cholesterol rich vesicles were more abundant in *LC3B*^-/-^ RPE compared to WT (**Supplementary Figure S3B**).

### Decrease in Pro-homeostatic Protective Docosanoid Mediators

**FIGURE 5:**
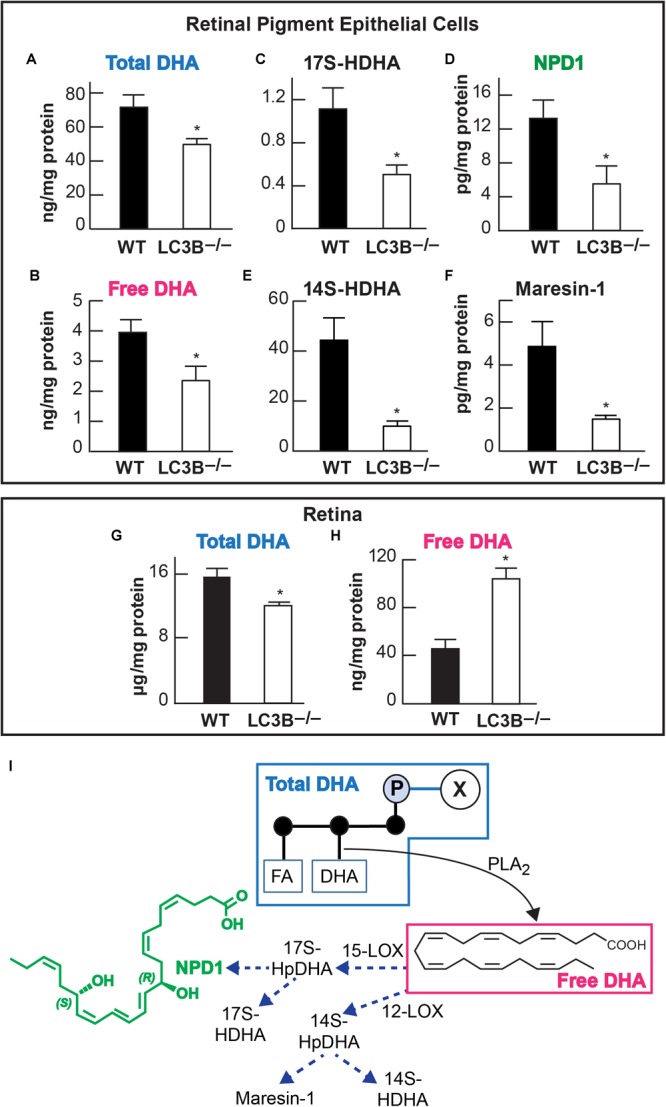
Relative abundance of DHA (docosahexaenoic acid) and of docosanoids in retinal pigment epithelial cells (RPE/choroid) and in retina of wild -type and *LC3B^-/-^*. In retinal pigment epithelial cells (RPE/choroid): Esterified DHA (total) **(A)**; free DHA **(B)**; 17(S)-HDHA **(C)**; NPD1 **(D)**; 14(S)-HDHA **(E)**, and Maresin-1 **(F)**. In retina total **(G)** and free **(H)** DHA. Data obtained by LC/MS/MS lipid profiling. ^∗^*p* < 0.050. Mice were 18–20 months old. The diagram **(I)** depicts a phospholipid hydrolyzed by a PLA2 that releases DHA. In turn two paths for the formation of docosanoids are illustrated, one leading to NPD1 and the other to maresin-1.

Accumulation of neutral lipids and decrease in ketogenesis in the *LC3B*^-/-^ RPE suggests an impairment in availability of free fatty acids. DHA is an OS lipid derived free fatty acid, which under physiological conditions, is recycled back to the inner segment of the photoreceptor cell (Gordon and Bazan, 1990; Rodriguez de Turco et al., 1991). There is a marked decrease in esterified DHA in membrane phospholipids (**Figure 5A**) and free DHA in the *LC3B*^-/-^ RPE (**Figure 5B**). Free DHA serves as a substrate for the synthesis of NPD1, synthesized by 15-lipoxygenase-1 (**Figure 5I**) via the short live intermediate 17(S)-HpDHA. We identified the stable derivative of this intermediate, 17(S)-HDHA as well as NPD1. Consistent with a decrease in DHA, there was also a concomitant decrease in 17(S)-HDHA and NPD1 in *LC3B*^-/-^ RPE (**Figures 5C,D**). DHA also serves as the precursor for the synthesis of 12-lipoxygenase-derived docosanoids, with potent anti-inflammatory and pro-resolving bioactivity known as the maresins (macrophage mediators in resolving inflammation). Maresin-1 levels were also decreased as well as its stable intermediate derivative, 14(S)-HDHA in the *LC3B*^-/-^ RPE of 18–20 months old mice as compared to WT (**Figures 5E,F**). In the retina, the level of total DHA in *LC3B*^-/-^ mouse is significantly lower than that of WT (**Figure 5G**), but there is an enhancement in the free DHA pool size (**Figure 5H**).

### Development of Pro-inflammatory Microenvironment in the *LC3B*^-/-^

**FIGURE 6:**
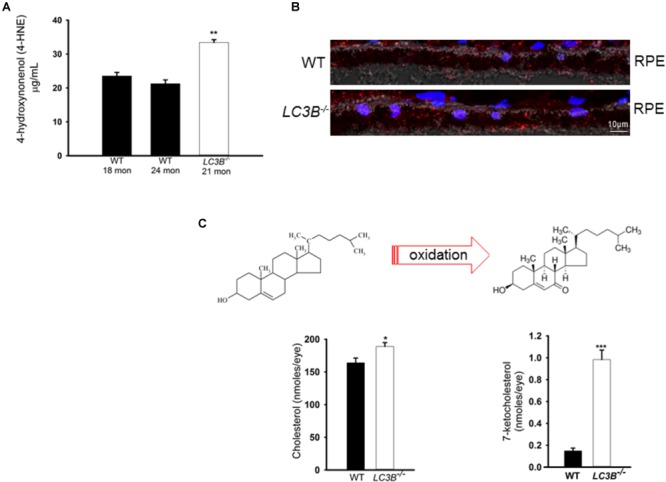
**(A)** 4-HNE-adducts and 7-ketocholesterol accumulates in the *L3CB^-/-^*. **(A)** Level of 4-hydroxynonenol protein adducts measured by ELISA for WT and *LC3B^-/-^* RPE/choroid explants (*N* = 6, ^∗∗^*p* < 0.002). **(B)** Confocal image showing immunostaining for 4-HNE adducts (red) in 20 months old WT and *L3CB^-/-^* RPE, nuclei (blue), Ch-choroid. **(C)** B Levels of cholesterol and one of its oxidation products, 7-ketocholesterol in WT and *L3CB^-/-^* RPE explants measured by LC/MS. Values are mean ± SEM from 4 individual mice (average age 16 months), ^∗^*p* < 0.050, ^∗∗^*p* < 0.002, and ^∗∗∗^*p* < 0.001.

Enhanced lipid accumulation in the oxidative stress environment of the RPE is predicted to result in enhanced lipid peroxidation. Lipid peroxidation levels were determined as 4-HNE-adducts by ELISA in aged mice. There was a ∼57% increase in levels of 4-HNE in the *LC3B*^-/-^ RPE explants as compared to controls (WT 24 months, *LC3B*^-/-^ 21 months *p* < 0.002, **Figure 6A**). Immuno-labeling with antibody to 4-HNE- adducts revealed 4-HNE localization to RPE and choroid (**Figure 6B**, 20 months WT and *LC3B*^-/-^) and substantial levels of 4-HNE were detected at 24 months for the *LC3B*^-/-^ (**Supplementary Figure S3C**, 27 months WT, 24 months *LC3B*^-/-^).

**FIGURE 7:**
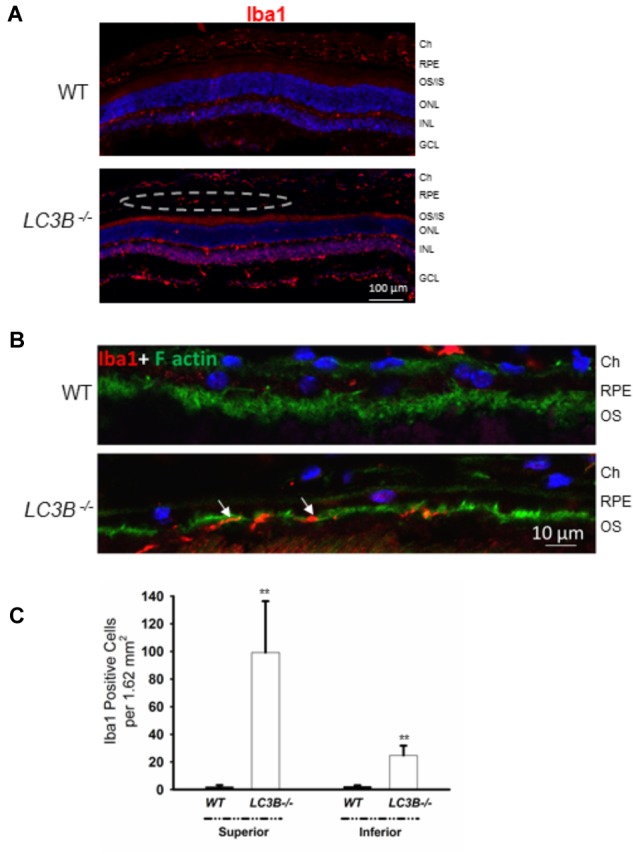
Loss of LC3B contributes to pro-inflammatory environment. **(A)** Immuno-staining of WT and *LC3B^-/-^* retinal sections with Iba1 showing numerous Iba1 positive cells (red, marked by dotted oval) in the *L3CB^-/-^* retina on the apical side of RPE, nuclei (blue); age 24 months. **(B)** Higher mag images showing the Iba1 positive cells (red) near the apical RPE, counterstained with phalloidin (green) to label the actin cytoskeleton; age ∼30 months. **(C)** Number of Iba1 positive cells per field in the superior and inferior RPE. Values are mean ± SEM from 4 individual mice (∼18 months), ^∗∗^*p* < 0.050.

A consequence of prolonged oxidative stress is the non-enzymatic conversion of cholesterol to the oxy-sterol, 7-ketocholesterol. While cholesterol levels in the *LC3B*^-/-^ RPE were elevated, perhaps more striking was the ∼5-fold increase in 7-ketocholesterol (7KCh) compared to WT RPE (**Figure 6C**). A consequence of elevated 7KCh in the retina is activation of microglia and their migration to the outer retina (Gupta et al., 2003; Indaram et al., 2015). To examine the pro-inflammatory role of 7KCh in the *LC3B*^-/-^, retinal sections were immunolabeled for a microglia/macrophage marker, Iba1. Interestingly, while there were several Iba1 positive cells in both WT and *LC3B*^-/-^ retina, these cells were more abundant in the sub-retinal space of *LC3B*^-/-^ mice (**Figures 7A,B**), clustered close to the optic nerve with 62 ± 20.19 vs. 2.13 ± 0.55 per 1.62 mm^2^ in the *LC3B*^-/-^ vs. WT, respectively (**Supplementary Figure S4A**). Sub-retinal Iba1 positive cells were observed in both superior and inferior retina, with ∼4-fold more Iba positive cells in the superior than inferior retina in the *LC3B*^-/-^ (**Figure 7C, Supplementary Figure S4B**, and see **Supplementary Figure S4C** for no anti-Iba1 control). There appears to be increase in Iba positive cells in the inner retina of *LC3B*^-/-^ compared to the WT, and may represent migrating activated microglia (Kim et al., 2014).

### Visual Function Loss in *LC3B*^-/-^

**FIGURE 8:**
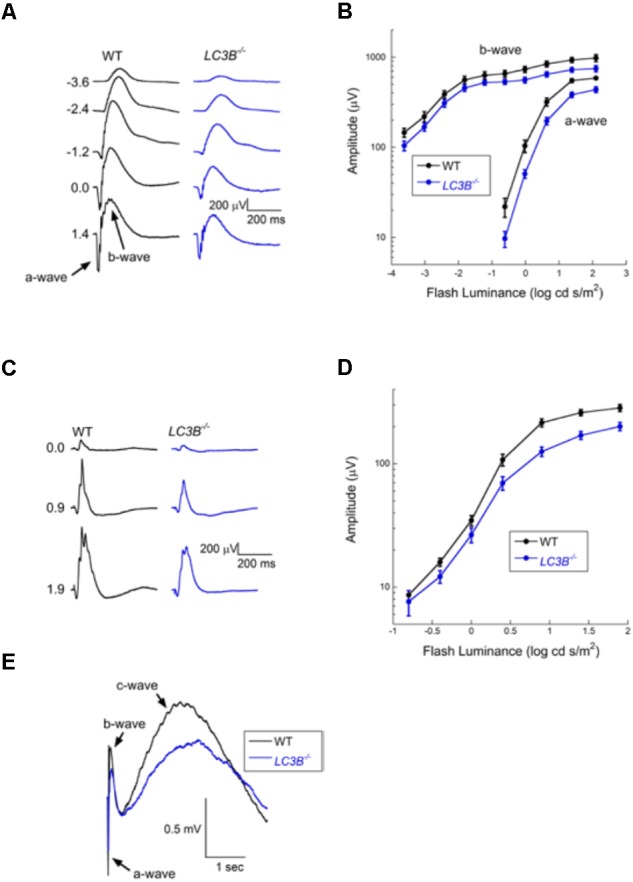
Electroretinographic comparison of WT and *L3CB^-/-^* mice (age 14–20 months). **(A)** Representative dark-adapted ERGs obtained from a WT (left) and a *L3CB^-/-^* (right) mouse to strobe flash stimuli presented to the dark-adapted eye. Flash luminance (in log cd s/m^2^) is indicated to the left of each pair of waveforms. **(B)** Amplitude of the major ERG components plotted against flash luminance. Data points indicate average (±SEM) of 7 mice. **(C)** Representative light-adapted ERGs obtained from a WT (left) and a *L3CB^-/-^* (right) mouse to strobe flash stimuli superimposed on a steady adapting field. Flash luminance (in log cd s/m^2^) is indicated to the left of each pair of waveforms **(D)** Amplitude of the light-adapted ERG plotted against flash luminance. Data points indicate average (±SEM) of 7 mice. **(E)** Representative c-waves obtained from a WT (left) and a *L3CB^-/-^* (right) mouse to a 1.4 log cd s/m^2^ flash presented to the dark-adapted eye. The c-wave peaks after the b-wave, and is reduced and delayed in *L3CB^-/-^* as compared to WT mice.

Defects in RPE-Photoreceptor phagocytosis prompted us to evaluate retinal function. A series of ERGs obtained from a representative WT and *LCB3*^-/-^ mouse are compared in **Figure 8A**. These responses were obtained to stimuli presented to the dark-adapted eye, providing an analysis of the rod pathway. The waveform of the *LCB*^-/-^ ERGs are comparable to those of WT, except for an overall reduction in response amplitude. In **Figure 8B** we compare the luminance-response functions for the major components of the dark-adapted ERG, the a-wave reflecting the mass light-induced closure of channels along the rod outer segment (Lamb and Pugh, 2006) and the b-wave reflecting the mass response of rod depolarizing bipolar cells following synaptic transfer of the photoreceptor signal (Hood and Birch, 1996; Robson and Frishman, 1996). Throughout the stimulus range examined, responses of *LC3B*^-/-^ mice were reduced as compared to those of age-matched WT animals. On a log amplitude scale, this reduction is seen to be relatively consistent across flash luminance. ANOVA analysis showed that the reduction was significant for the a-wave (*P* < 0.0001) and b-wave (*p* < 0.0001). To further evaluate these reductions, we fit Equations (1) and (2) to the leading edge of the a-wave and the b-wave luminance-response function, respectively. For the a-wave, the amplitude parameter RmP3 was significantly reduced in *LCB3^-/-^* (mean ± sem: 401.0 ± 29.0 μV) as compared to WT (563.8 ± 27.0 μV) mice (*P* < 0.001) while neither A nor td varied between genotypes. For the b-wave, the amplitude parameter *R*_max_ was significantly reduced in *LCB3*^-/-^ (571.9 ± 39.1 μV) as compared to WT (724.4 ± 59.1 μV) mice (*p* < 0.04) while K did not differ between genotypes.

The overall waveform of the cone ERGs obtained from *LC3B*^-/-^ mice were comparable to those of WT, except for an overall reduction in response amplitude (**Figure 8C**). The luminance-response functions of the cone ERG for WT and *LC3B*^-/-^ mice were compared as shown in **Figure 8D**. The responses of *LC3B*^-/-^ mice are reduced as compared to WT (*P* < 0.0001). When the cone ERG luminance-response functions were fit by Equation (2), the amplitude parameter *R*_max_ was significantly increased in *LCB3*^-/-^ (211.1 ± 17.4 μV) as compared to WT (283.5 ± 17.1 μV) mice (*p* < 0.04) while *K* did not differ between genotypes.

The mouse ERG contains slower duration components that reflect the response properties of non-neuronal cells (Kinoshita and Peachey, 2018). Here we examined the c-wave which appears as a positive with a peak 1.5–2 s after a brief flash (Kinoshita and Peachey, 2018). Representative c-waves obtained to 1.4 log cd s/m^2^ flashes from *LC3B*^-/-^ and WT mice are shown in **Figure 8E**. In the *LC3B*^-/-^ mouse, the c-wave was significantly reduced in amplitude (*P* < 0.02). The c-wave reduction (to 68.9% of WT) is comparable to that of RmP3 (to 71.1% of WT). The c-wave peak time was also significantly slower in *LC3B*^-/-^ as compared to WT mice (*P* < 0.04). Taken together, in *LC3B*^-/-^ mice the c-wave amplitude reduction and delay are consistent with a reduced contribution of the positive signal generated by the RPE apical membrane, although other mechanisms are possible.

**FIGURE 9:**
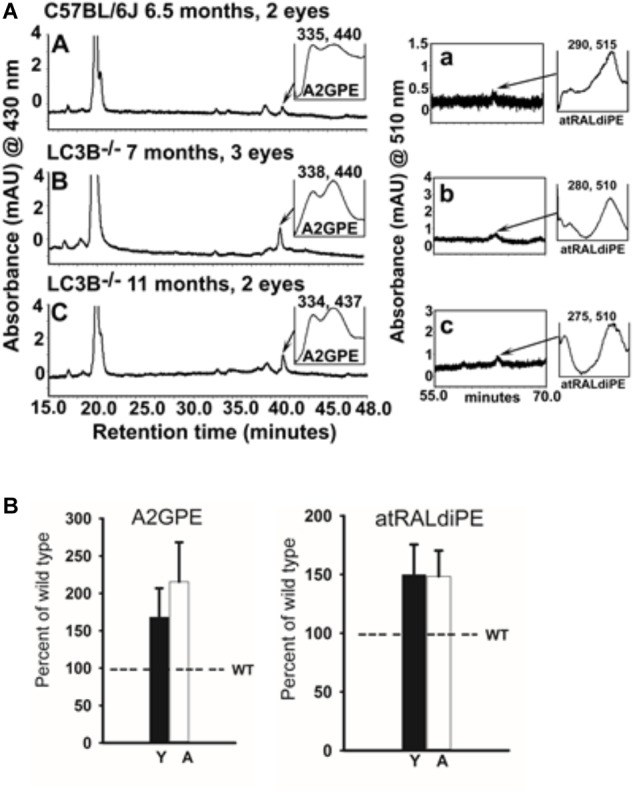
**(A)** Bisretinoid accumulate in the *LC3B^-/-^* mouse. Left, representative chromatogram at 15–48 min retention time (monitored at 430 nm) for WT (6.5 months) and *LC3B^-/-^* (7 and 11 months old) mice showing UV visible absorption spectrum of A2GPE; Right, chromatograms at 55–70 min retention time with monitored at 510 nm showing absorbance spectrum of atRALdiPE. *mAU*, milli-absorbance unit. **(B)** Quantification of the lipofuscin pigments A2GPE (*left*) and atRALdiPE (*right*) in the eyes WT (6.5 months) and *LC3B^-/-^* (7 and 11 months). Levels were determined as integrated peak areas normalized to synthesized external standards. Values (mean ± SE) are expressed as percentage of WT; Y (young), A (aged).

Bisretinoids, adducts of retinaldehyde form in the photoreceptor cells and accumulate in RPE as lipofuscin (Ueda et al., 2016). To assess the involvement of LC3B-dependent processes on bisretinoid accumulation, WT and *LC3B*^-/-^ eye-cups were analyzed for levels of bisretinoid pigments, specifically, all-*trans*-retinal dimer-phosphatidylethanolamine (atRALdiPE) and A2-glycero-phosphoethanolamine (A2GPE). The levels of these adducts were over twofold higher in *LC3B*^-/-^ compared to WT mice (**Figures 9A,B**).

## Discussion

In terminally differentiated cells, autophagy plays a critical role in intracellular quality control. The inability to divide predisposes these cells to the accumulation of cellular waste, contributing to increased oxidative stress and inflammasome activation (Handa et al., 2017). Autophagic processes are protective; ridding cells of defective mitochondria, peroxisomes and aggregated proteins. Here we examine the consequences of defective LC3B mediated autophagy. The present study identifies the LC3 isoform, LC3B, as a critical determinant of lipid homeostasis thereby as essential in regulating the inflammatory state of the retina/RPE. Although the mouse retina and RPE also express LC3A, there appears to be little to no compensatory upregulation in this isoform (**Figure 1**). Nor does LC3A functionally compensate for LC3B in RPE as it relates to phagosome degradation and lipid metabolism with altered lipid homeostasis over time. Moreover, there is no gene corresponding to *MAPILC3C* in mouse and in human LC3C is not expressed in the RPE (Dhingra et al., 2018). Collectively, these studies suggest that loss of LC3B leads to age-dependent lipid accumulation due to a lifetime of delayed phagosome maturation and content degradation, as described below.

### Consequences of Dysregulated Lipid Homeostasis

Retinal pigment epithelium cells are functionally distinct from most other phagocytes in that they must ingest OSs daily, thus any inefficiency in daily phagosome degradation contributes to pathogenesis and age-related visual impairment. In this study we found that the *LC3B^-/-^* mouse manifests slowed phagosome degradation, accumulation of RPE and sub-RPE lipid deposits. Excessive lipid accumulation and/or formation of lipid-peroxidation adducts in RPE/Ch as observed in the *LC3B^-/-^* model has also been described in mice with an RPE specific loss of Atg7 or Atg 5 (Zhang et al., 2017) as well as in the kinesin-1 light chain (KLC1) KO mouse (Jiang et al., 2015). Dysregulation of RPE lipid homeostasis is particularly detrimental because RPE are at a high risk for uncompensated oxidative stress (UOS), characterized by persistent oxidative stress with levels of reactive oxygen species (ROS) exceeding the capacity of the antioxidant enzymes to handle it. These cells constantly manage ROS, resulting from an oxygen-rich environment, the photoreactivity of bisretinoid lipofuscin (Zhou et al., 2005; Zhou J. et al., 2015), high metabolic activity, and daily high flux of polyunsaturated fatty acids from OS phagocytosis as well as exposure to the oxidizing effects of blue light (Cuervo, 2004; Asatryan and Bazan, 2017; Handa et al., 2017). In the absence of LC3B, the undigested lipids serve as substrates for peroxidation reactions with elevated levels of 4-HNE lipid peroxidation adducts, which accumulate in the RPE and choroid (**Figure 6**). Moreover, experiments using mouse and *in vitro* models have demonstrated associations between RPE bisretinoid lipofuscin, the generation of ROSs and the oxidation of lipid and protein cellular substrates (Zhou et al., 2005; Zhou J. et al., 2015). Lipid oxidation is a critical factor in the development of AMD (Balaratnasingam et al., 2017; Golestaneh et al., 2017; Handa et al., 2017; Xu et al., 2017).

Dysregulation of cholesterol metabolism and oxysterol accumulation are also associated with AMD as well as other pathologies involving oxidative stress such as Huntington’s disease, atherosclerosis, and cystic fibrosis (Iuliano et al., 2003, 2009; Jenner et al., 2007; Kreilaus et al., 2016). We examined, the levels of 7-ketocholesterol, a pro-inflammatory oxysterol formed by the auto-oxidation of cholesterol and cholesterol esters, compounds that accumulate in Bruch’s membrane as a consequence of aging (Curcio, 2011; Pikuleva and Curcio, 2014). Biochemical analysis pointed to a ∼5-fold increase in 7KCh in *LC3B^-/-^* RPE/Ch compared to WT. We observed a similar accumulation of filipin-positive cholesterol vesicles and neutral lipids in the *LC3B^-/-^* RPE/Ch (**Figure 4C** and **Supplementary Figure S3B**). These results are particularly significant because to our knowledge, the *LC3B^-/-^* mouse is the first to show elevated 7KCh in ocular tissue, *in vivo*. Moreover, given that the RPE handles large amounts of lipids, with OSs serving as a major source of cholesterol, understanding how loss of LC3B contributes to the regulation of cholesterol efflux pathways (Storti et al., 2017) will provide valuable insight into age-related perturbations of lipid homeostasis.

Subretinal injection of 7KCh has been shown to induce migration of Iba1 positive microglia to the outer retina suggesting a role as a chemo-attractant and thus altering the immune environment (Indaram et al., 2015). In agreement with increases in 7KCh levels in *LC3B^-/-^*, we observed far more Iba1 positive cells in the central sub-retinal space in the *LC3B^-/-^* relative to the age- matched control mice (**Figures 7A,B**). These cells were more abundant in the superior retina, similar to that observed in mice lacking aryl hydrocarbon receptor (Ahr*^-/-^*) (Kim et al., 2014). Increase in number of sub-retinal microglia with age has been reported and more importantly is considered as a common hallmark of various retinal degenerative and inflammatory diseases (Karlstetter and Langmann, 2014). These include retinitis pigmentosa, late-onset retinal degeneration and AMD in human (Gupta et al., 2003; Indaram et al., 2015) as well as in mouse models of AMD (Combadiere et al., 2007). Although microglia are also neuroprotective under physiological conditions (Ramirez et al., 2017), it has been proposed that dysregulated overactive microglia, those clearing oxysterol debris by way of example, may contribute to the prolonged inflammation and chronic disease progression (Gao and Hong, 2008). Future studies are underway to determine the relationship between lipid accumulation and microglia recruitment.

Docosahexaenoic acid is an OS lipid derived free fatty acid, that under physiological conditions, is recycled back to the inner segment of the photoreceptor cell by the short loop via the interphotoreceptor matrix (Gordon and Bazan, 1990; Rodriguez de Turco et al., 1991). At the onset of disruptions of homeostasis DHA is channeled toward the synthesis of NPD1 and of other docosanoids (Bazan, 2007). In the absence of LC3B, the biosynthesis of the docosanoids, NPD1 and maresin-1, is attenuated, likely further exacerbating homeostatic disruptions and the pro-inflammatory environment. The RPE displays decreased abundance of docosahexaenoyl containing phospholipids (**Figure 5G**) reflecting an impairment in the shedding/phagocytosis and/or in the handling of this disk membrane components for proper photoreceptor outer segment renewal. The biosynthesis of the mediator made on demand to cope with homeostasis disruptions, NPD1 is in fact downregulated (Bazan, 2007).

The traditional view of β-HB as solely an energy carrier has greatly expanded to include β-HB mediated inhibition of inflammasome activation (Youm et al., 2015). Prolonged fasting reduces inflammation, with β-HB selectively suppressing inflammasome activation in response to lipotoxic fatty acids (Vasconcelos et al., 2014). This inhibitory effect is independent of autophagy, and TCA cycle dependent oxidation, with other ketogenic intermediates such as acetoacetyl-CoA having no affect. Herein we show that β-HB production due to slowed OS clearance is delayed in the *LC3B^-/-^* RPE, with maximal β-HB release seen 6 h after light onset, in contrast to WT, where maximal release is at 2 h after light onset (Reyes-Reveles et al., 2017). Moreover, total β-HB release is decreased, suggesting a further loss of protective lipid-derived mediators likely contributing to the RPE and retinal abnormalities observed in aged mice.

In response to oxidative stress, autophagy increases in an attempt to remove oxidatively damaged organelles (Lee et al., 2012; Seo et al., 2012). In the *LC3B^-/-^* mouse we observe a breakdown in protective role(s) of autophagy over time; the RPE shows signs of oxidative stress, the accumulation of 4-HNE-adducts both in the RPE and sub-RPE space leading to loss of RPE and retinal function. The photooxidative processes initiated by PR derived bisretinoids likely also contribute to HNE-modifications (Zhou et al., 2005) and trapping of cholesterol within RPE, potentially altering autophagic processes (Toops et al., 2015). Since bisretinoid form in photoreceptor cell outer segments before phagocytosis by RPE, the increase in bisretinoids observed may be due to alterations in PR lipid homeostasis in the absence of LC3B. It is intriguing to consider that while loss of LC3B over time leads to AMD-like pathogenesis, the defects are not observed in younger mice, suggesting that LC3A or other non-autophagy associated degradative processes contribute to phagosome degradation and organelle maintenance.

It is important to point out that the most-often studied endogenous autophagic marker is LC3B. A caveat to such studies is that a substantial majority of these studies use anti-LC3 antibodies presumed to be LC3B specific. Here we show that a rabbit anti-LC3B (D11, from Cell Signaling), specifically detects LC3B, while, rabbit anti-LC3A (ab62720, Abcam) detects LC3A. Specifying individual LC3 isoform is necessary as the isoforms have different expression patterns and biology (Cann et al., 2008; Koukourakis et al., 2015).

In the present studies we used a global *LC3B^-/-^* mouse, but we reason that the retinal phenotype seen is largely due to loss of LC3B in retina and RPE and not due to systemic effects. This is based on a considerable body of evidence from earlier studies using *LC3B^-/-^* mouse and our work: (1) *LC3B^-/-^* mice develop normally, reach adulthood, are fertile, in contrast to mice with global KOs of the other autophagy related genes like *Atg5* and *Atg7* that die within a day after birth (Kuma et al., 2004; Komatsu et al., 2005; Cann et al., 2008). This may be due to functional redundancy between LC3B and other Atg8 homologs (e.g., GABARAP) (Cann et al., 2008; Klionsky et al., 2016). (2) There is no indication of systemic defects in the 4 previous studies using this mouse (Cann et al., 2008; Chen et al., 2010; Lee et al., 2011; Reed et al., 2015). (3) Role of LC3B in lungs has been uncovered under diverse stimuli: it has been shown to play a protective role in hypoxia; pro-pathogenic role in cigarette smoke induced emphysema; increased susceptibility to lung pathology during respiratory viral infection. These conditions are unlikely to be pertinent to our current study (Chen et al., 2010; Lee et al., 2011; Reed et al., 2015). For example, *LC3B^-/-^* mice display enhanced indices of pulmonary hypertension after chronic hypoxia but not under normoxic conditions (Lee et al., 2011). These results are relevant as in our own studies LC3B is protective during aging/increased oxidative stress as its loss leads to several retinal/rpe abnormalities. (4) Moreover, *LC3B^-/-^* mice are same weight as age-matched wild type from 2 to 18 months of age (**Supplementary Figure S4D**), they live for over 2 years, and are overall healthy like WT controls.

### Importance of LC3B for Efficient OS Degradation and Lipid Metabolism

The processes of OS phagocytosis and lipid metabolism rely on two different aspects of autophagy and LC3 function. During OS phagocytosis, LC3B associates with ingested phagosomes targeting them for degradation in a process known as LAP (Kim et al., 2013; Frost et al., 2014, 2015, 2017; Muniz-Feliciano et al., 2017). This process requires the LC3B adapter, melanoregulin (MREG) to mediate LC3B association with newly ingested OS-phagosomes (Frost et al., 2015). Phagosome accumulation is observed in the absence of MREG (Damek-Poprawa et al., 2009) as well as in the absence of LC3B in the current studies (**Figure 4A**). Free fatty acids generated upon OS digestion serve as substrates for mitochondrial FAO and ketogenesis (Reyes-Reveles et al., 2017). The metabolic fate of the free fatty acids relies on the maintenance of healthy mitochondria and peroxisomes, organelles necessary in the oxidation of long chain fatty acids. The RPE utilizes LC3 in both processes; in LAP to decorate the outer membrane of phagosomes and in mitophagy and pexophagy to remove defective mitochondria and peroxisomes, respectively (Ferrington et al., 2016). Pexophagy and mitophagy both utilize the LC3 adapter p62, thus the cytoprotective role of autophagy under stress and increased p62 likely favors these two LC3-dependent functions (Mitter et al., 2012; Wang et al., 2014; Song et al., 2017).

Our current studies suggest that in the absence of LC3B, it is the inability to degrade ingested OS lipids that contributes to RPE abnormalities given that phagosome levels do not return to baseline (**Figure 4A**) and there is an accumulation of lipid deposits (**Figure 4C** and **Supplementary Figure S3**), concurrently, citrate synthase activity (mitochondrial TCA cycle) is unaltered (**Supplementary Figure S1D**). Moreover, p62 levels in the *LC3B^-/-^* RPE in both young and older mice are unchanged suggestive of relatively intact selective autophagy. Given the temporal distribution of LC3B and the timing of OS phagocytosis, we predict that upon light onset LAP-dependent pathways predominate, consistent with high MREG-LC3B levels at this time (Frost et al., 2015). Whereas, after the bolus of ingested OS lipids is metabolized, the maintenance of damaged mitochondria and/or peroxisomes through selective autophagy predominates; in fact, there is a second LC3 peak 6 h after light onset (Yao et al., 2014; Frost et al., 2015). Consistent with this interpretation recent studies have also shown that LAP-mediated increases in RUBCN inhibit starvation induced autophagy in the RPE (Muniz-Feliciano et al., 2017). Whether RUBCN also similarly modulates mitophagy is an unresolved question; further studies to understand the specific role of LC3A in retina and RPE may address such issues.

### Autophagy in Age-Related Retinal Disease – New Insights

Numerous reviews posit a role for autophagy in aging and age-related macular degeneration (AMD) (Mitter et al., 2012; Kaarniranta et al., 2013; Kaarniranta et al., 2017). RPE cultures derived from human donor eyes (patients with AMD), exhibited increased intracellular lipid accumulation and oxidative stress. LC3II/LC3I ratios were lower in RPE from AMD patients with decreased autophagic flux and mitochondrial accumulation (suggestive of altered mitophagy) (Golestaneh et al., 2017), leading these and other investigators doing *in vitro* work to conclude that dysregulated or dysfunctional autophagy contributes to AMD. The studies presented herein are consistent with this premise, however, they also highlight the complexity associated with autophagic process. LC3B or LC3A is required for starvation induced as well as selective autophagy which includes, pexophagy, mitophagy, secretory autophagy, LAP as well as chaperone mediated autophagy, among others. In light of our studies and the rapidly growing body of evidence detailing specific autophagy associated process, the use of “autophagy” as an all-encompassing umbrella is likely a misnomer. Understanding the relationship between specific LC3-dependent processes, specific LC3 isoforms, spatial expression and the temporal regulation of these components and associated pathways is critical to our understanding of “autophagic”- dysregulation and disease. The visual system is unique in that in these terminally differentiated cells, LC3 levels fluctuate in circadian pattern. This fluctuation is likely correlated with RPE and PR physiological function, for example recent studies suggest that OS phagocytosis triggers a downregulation of starvation induced autophagy so that LC3B-associated phagocytosis may proceed (Muniz-Feliciano et al., 2017). The *LC3B^-/-^* mouse has and will continue to provide a valuable model in which this relationship can be uncoupled thus aiding in the design of targeted therapeutics.

## Author Contributions

KB-B, NJP, NB, JS, NSP, BB, and AD designed the research and wrote the paper. AD, JR-R, RS, BJ, LD, and HK performed the research and analyzed the data. BB and NSP performed the non-invasive imaging analyses and functional studies and analyzed the data. The JS lab performed the bisretinoid analysis and analyzed the data. The NB lab performed the lipidomics analyses and analyzed the data. All authors read and approved the manuscript.

## Conflict of Interest Statement

The authors declare that the research was conducted in the absence of any commercial or financial relationships that could be construed as a potential conflict of interest.

## Supplementary Material

FIGURE S1 **(A)** Representative immunoblots showing expression of LC3B in the RPE (using a second LC3-B antibody, D11) from 4, 12, 16, and 20 months old mice. **(B)** Representative immunoblot showing levels of HMGCS2 in *LC3B^-/-^* and WT RPE. **(C)** Mean (±SEM) HMGCS2/actin levels in *LC3B^-/-^* and WT RPE. **(D)**. Citrate synthase activity in RPE explants from *LC3B^-/-^* and WT RPE isolated at 7 am. Data **(C,D)** represents 3 mice (6-eyes) of 12–15 month old animals.

FIGURE S2 Electron micrographs from ∼4 months old WT and *LC3B^-/-^* retina at 7:30 am (30 min after light onset) and 2 pm (7 h after light onset), showing phagosome accumulation (arrows).

FIGURE S3 **(A)** Representative confocal image of neutral lipid deposits detected by staining with BODIPY^493/503^ in WT and *LC3B^-/-^* mouse RPE (2 and 18 months). **(B)** Confocal image showing filipin staining in RPE (left), low magnification image (right) from WT and *LC3B^-/-^* (18 months) mice. **(C)** Confocal image showing 4-HNE immunostaining of WT (27 months) and *LC3B^-/-^* (24 months) retina.

FIGURE S4 **(A)** Confocal images of RPE flat mount from 18 months old WT (left) and *LC3B^-/-^* (right) immuno-stained for Iba1 (red), F-actin (green). **(B)** Confocal images of superior (left) and inferior (right) areas of RPE flat mounts from *LC3B^-/-^* immuno-stained for Iba1 (red), F-actin (green) (age ∼18 months). **(C)** Representative confocal images of WT and *LC3B^-/-^* retina immuno-stained for rhodopsin (4D2, green) and no primary control for Iba 1 (secondary: anti rabbit IgG Alexa Fluor 594, red). OS, outer segments; ONL, outer nuclear layer; INL, inner nuclear layer). **(D)** Bar graph showing mean weight of age-matched WT and *LC3B^-/-^* mice at 2, 7.5, and 18 months. The weights of 10 mice per age were measured and represent an equal number of male and female mice.
