# Supplementary figures and images for: Microtubule-Associated Protein 1 Light Chain 3B, (LC3B) Is Necessary to Maintain Lipid-Mediated Homeostasis in the Retinal Pigment Epithelium
Source: Front Cell Neurosci. 2018 Oct 8;12:351. doi: 10.3389/fncel.2018.00351 (PMC6186781; doi:10.3389/fncel.2018.00351)

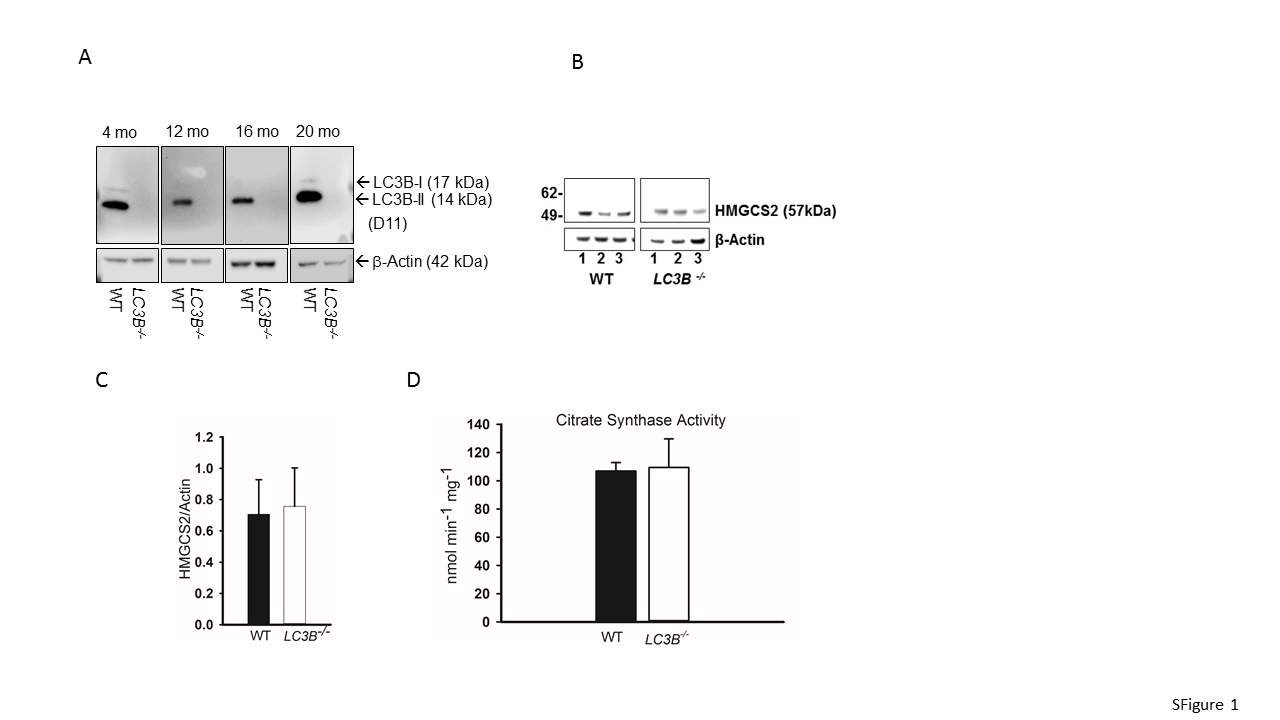

Supplement: FIGURE S1 — (A) Representative immunoblots showing expression of LC3B in the RPE (using a second LC3-B antibody, D11) from 4, 12, 16, and 20 months old mice. (B) Representative immunoblot showing levels of HMGCS2 in LC3B-/- and WT RPE. (C) Mean (±SEM) HMGCS2/actin levels in LC3B-/- and WT RPE. (D). Citrate synthase activity in RPE explants from LC3B-/- and WT RPE isolated at 7 am. Data (C,D) represents 3 mice (6-eyes) of 12–15 month old animals. [file Image_1.TIF]

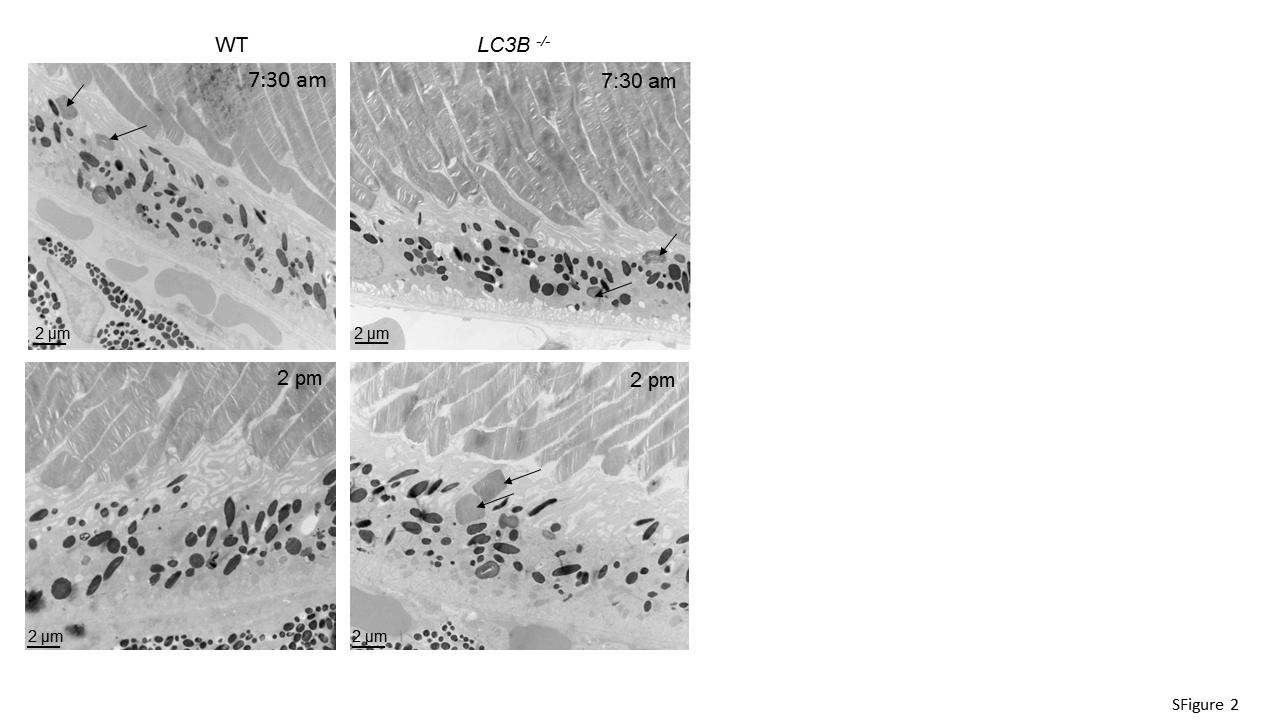

Supplement: FIGURE S2 — Electron micrographs from ∼4 months old WT and LC3B-/- retina at 7:30 am (30 min after light onset) and 2 pm (7 h after light onset), showing phagosome accumulation (arrows). [file Image_2.TIF]

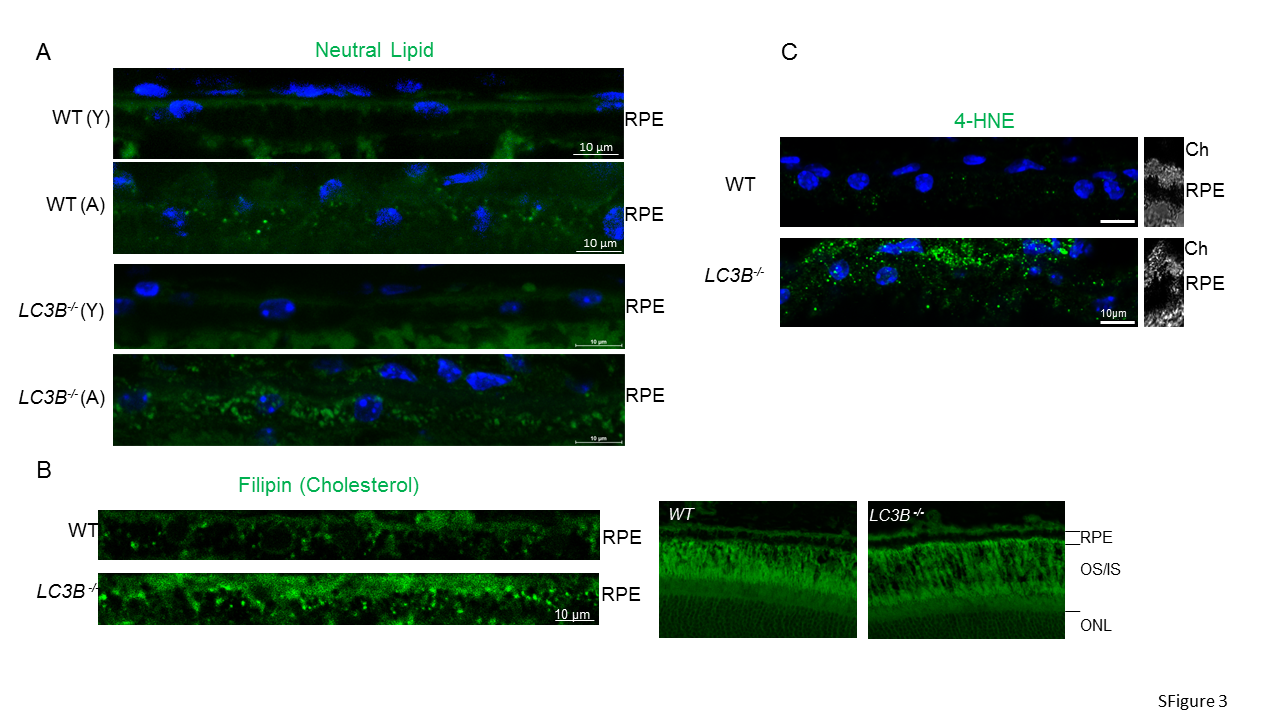

Supplement: FIGURE S3 — (A) Representative confocal image of neutral lipid deposits detected by staining with BODIPY493/503 in WT and LC3B-/- mouse RPE (2 and 18 months). (B) Confocal image showing filipin staining in RPE (left), low magnification image (right) from WT and LC3B-/- (18 months) mice. (C) Confocal image showing 4-HNE immunostaining of WT (27 months) and LC3B-/- (24 months) retina. [file Image_3.TIF]

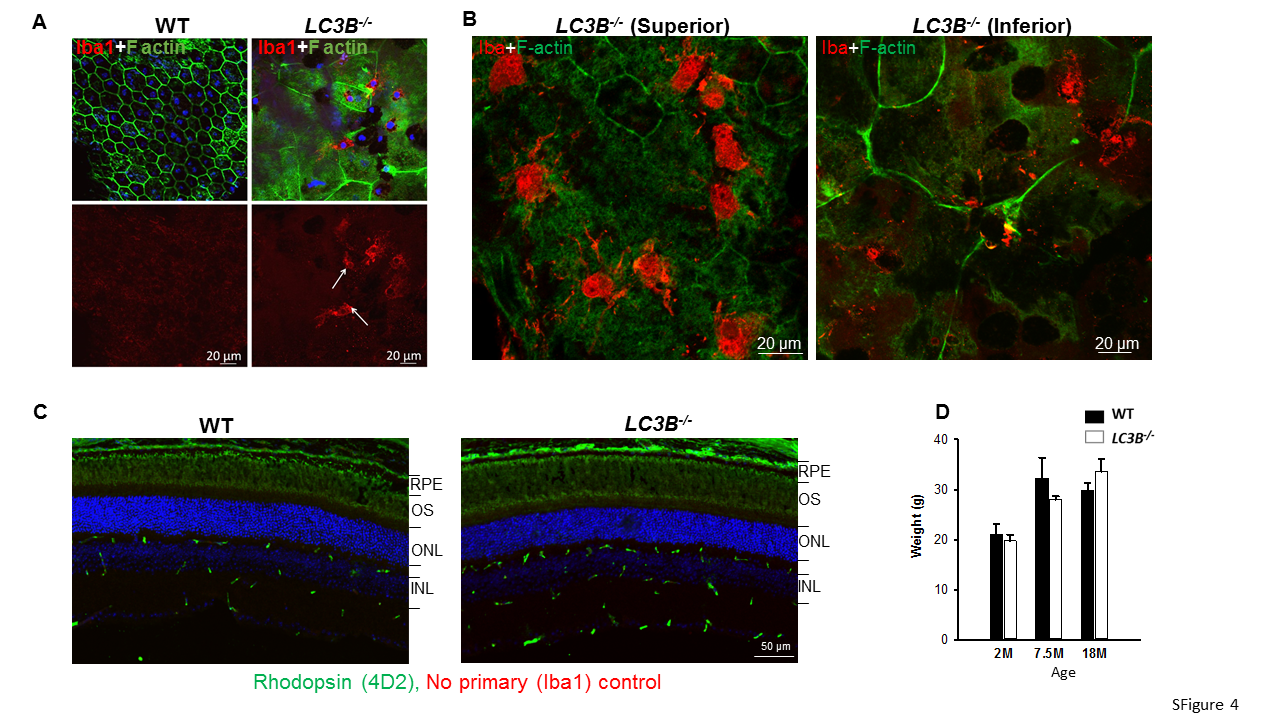

Supplement: FIGURE S4 — (A) Confocal images of RPE flat mount from 18 months old WT (left) and LC3B-/- (right) immuno-stained for Iba1 (red), F-actin (green). (B) Confocal images of superior (left) and inferior (right) areas of RPE flat mounts from LC3B-/- immuno-stained for Iba1 (red), F-actin (green) (age ∼18 months). (C) Representative confocal images of WT and LC3B-/- retina immuno-stained for rhodopsin (4D2, green) and no primary control for Iba 1 (secondary: anti rabbit IgG Alexa Fluor 594, red). OS, outer segments; ONL, outer nuclear layer; INL, inner nuclear layer). (D) Bar graph showing mean weight of age-matched WT and LC3B-/- mice at 2, 7.5, and 18 months. The weights of 10 mice per age were measured and represent an equal number of male and female mice. [file Image_4.TIF]
